# Inert nanoparticle suppression of gas explosion in the presence of obstacles

**DOI:** 10.1039/c8ra06000b

**Published:** 2018-11-22

**Authors:** Xiaoping Wen, Tengfei Su, Fahui Wang, Haoxin Deng, Kai Zheng, Bei Pei

**Affiliations:** School of Mechanical and Power Engineering, Henan Polytechnic University Jiaozuo 454003 PR China wenxiaoping666@163.com; State Key Laboratory of Coal Mine Disaster Dynamics and Control, Chongqing University Chongqing 400044 PR China; State Key Laboratory Cultivation Bases Gas Geology and Gas Control, Henan Polytechnic University Jiaozuo Henan 454003 PR China

## Abstract

The suppressing effects of inert nanoparticles on methane–air explosion, in an obstructed chamber with internal dimensions of 150 mm × 150 mm × 500 mm, were experimentally investigated. To this end, the flame behaviors in the presence of obstacles as well as overpressure transients during the explosions with and without nanoparticles were compared. Additionally, the effects of density, diameter, and material of nanoparticles on the suppressing behaviors were analyzed as well. The results showed that the methane–air deflagrating flame remains generally light blue if the nanoparticles are added. In particular, the flame obstacle interaction may enhance the suppression effect of the nanoparticles, and the flame acceleration rate and the peak overpressure decrease significantly. Increasing explosion suppression is seen up to about 100 g m^−3^ particle density, but further increase in particle density, up to 150 g m^−3^, yields no further increase in the explosion suppression ability. And as the particle size decreases, the suppressing effect is more evident. The experiments also showed that Al(OH)_3_, Mg(OH)_2_, and SiO_2_ all can be used to suppress the flame propagation and overpressure. However, the metal hydroxides suppress the methane explosions even more efficiently than SiO_2_ particles; Al(OH)_3_ particles have a slightly better inhibiting effect than Mg(OH)_2_. Mechanisms for the observed phenomena were discussed.

## Abstract 2

The suppressing effects of inert nanoparticles on methane–air explosion, in an obstructed chamber with internal dimensions of 150 mm × 150 mm × 500 mm, were experimentally investigated.

## Graphical abstract

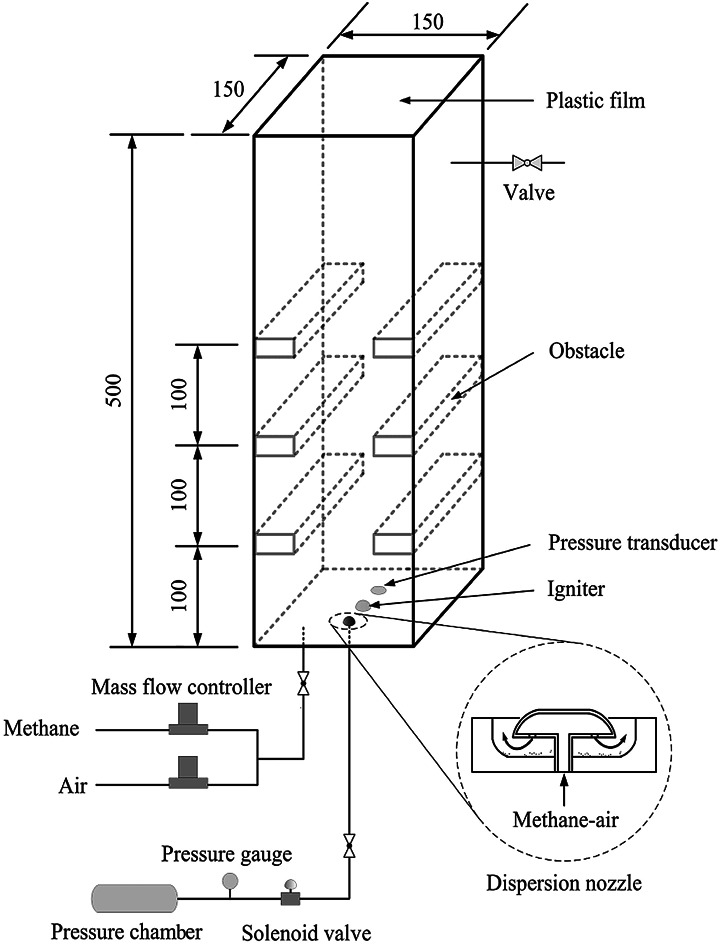

## 1 Introduction

Gas explosion is one of the major disasters in coal mines, which always causes large losses and serious damage. Past loss experiences in China showed that prevention of gas explosion only by reducing the risk of accidental releases, formation of flammable clouds, and ignition is not sufficient.^1,2^ Therefore, we have to build in protective barriers against gas explosion in our facilities. In the past decades, a considerable amount of experimental and theoretical study on explosion suppression technology has been conducted.^3–11^ The inert particle barrier is one of the most promising techniques for suppressing such incidents. The technique is based on mounting shelves under the roof in galleries, where the inert dust is distributed. In the case of gas explosion, some inert fine particles sprayed over the flame profile can absorb the explosion wave energy and extinguish flame propagation.

Previous studies have indicated that the inert particles can directly decrease the explosion pressure, and the particle size plays an important role in explosion suppression.^12–20^ From the literature, it is known that, for fixed particle density, more intense suppression is obtained for smaller particles. So far, due to the complicated process of explosion suppression, literature studies concerning the evaluation of explosion suppression using powders were essentially carried out on micro-sized particles,^21^ and thus there is little information concerning nanoparticle suppression characteristics. Actually, nanoparticles have higher specific surface area and are easy to contact with and absorb free radicals near the combustion reaction region, which directly reduces the combustion reaction intensity and in turn depresses the explosion flame speed and pressure wave. Therefore, the study of nanoparticle suppression of gas explosion is important for both scientific research and practical applications.

Another interesting issue is the analysis of the physics of the interactions between explosion flame and particles, and between the flame and obstacles. In real gas explosion, the flame front away from an ignition source may encounter obstacles along its path. It is generally accepted that the burning rate of a propagating flame is enhanced when it interacts with solid obstacles,^22–28^ which may influence the explosion itself and affect the explosion prevention efficacy, that is to say, the obstacles will also result in the inert particles suppressing behaviors of gas explosion more complex. The understanding of the processes is necessary to correctly predict the course of explosions and their mitigation. However, few studies exist in the literature related to the mitigation by inert particles of gas explosions with consideration of obstacles.

In the present work, we experimentally compared in detail flame behaviors as well as overpressure transients with and without nanoparticles, and explored the interaction between flame and inert nanoparticles during gas explosion suppression in an obstructed chamber, which aimed to reveal the effects of presence of obstacles. Additionally, the effects of density, diameter, and material of nanoparticle on the suppressing behaviors for gas explosion were also discussed.

## 2 Experimental apparatus and procedures

**Fig. 1:**
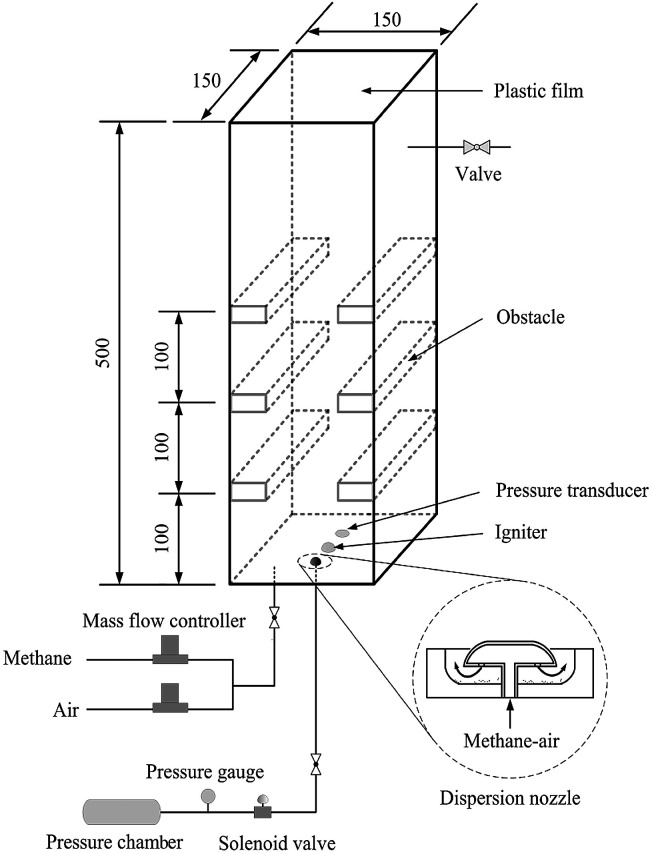
Schematic of experimental setup.

Experimental apparatus consists of an explosion chamber, a gas supply system, a dusting system, a data acquisition system, a control unit and an ignition system. The schematic diagram of the setup is shown in Fig. 1. The experimental chamber, with dimensions of 150 × 150 × 500 mm, is constructed with 20 mm-thick perspex to facilitate the application of optical diagnostics. It is open at top end and closed at the bottom. A thin plastic film is used to seal the open end in order to contain the flammable mixture before ignition. On both the left and right walls of the chamber, three pairs of solid steel obstacles (37.5 × 150 × 10 mm) are fixed at 100 mm spacings from the bottom end thus giving an overall blockage ratio of 50%. The dusting system consists of a pressure vessel, a solenoid valve and a dispersion nozzle. The nozzle mounted close to the center of the bottom plate position of the chamber is controlled by the solenoid, which is commanded by the control unit. The nozzle structure is also shown in Fig. 1, which corresponds to the one used in the Hartmann tube.^29^

For each explosion experiment, the stoichiometric mixture of methane and air is filled into the modified Hartmann chamber through the bottom plate and may be vented through a valve positioned near the top of the perspex wall. The methane–air mixture is obtained by using two mass flow controllers. The flow-rate of the premixed mixture is about 5 L min^−1^, and this process continues for 10 minutes supplying a total of 50 liters of methane–air mixture which is more than 4 times the volume of the chamber. This step is necessary for purging the chamber and ensuring that the mixture in the test unit is homogeneous.^30^ Then the flow is stopped, and a certain mass of nanoparticles, which are initially placed in a stainless-steel cup, may be dispersed through the nozzle into the explosion chamber by the pressurized methane–air gases in the pressure vessel. During this process, the dust is dispersed into the chamber and distributed to form uniform dust cloud in the chamber. The dust dispersing structure is designed according to the Hartmann based experimental equipment commonly used in the dust explosion studies.^9,29^ The pressure used to disperse suppressing particles is 800 kPa.^31^ This pressure vessel is very small, so the initial pressure on the explosion chamber is relatively small. The pressurized mixture with the methane concentration of 9.5% are produced using the partial pressure method. Because the obstruction in the chamber has a turbulent excitation effect on the airflow, the nanoparticles can disperse more uniformly in the chamber. After 5 s delay, the mixture in the explosion chamber is ignited by a spark plug positioned at the center of the chamber's bottom plate.

The dynamic overpressure generated from the methane–air explosion is monitored at 5 kHz using a Keller type PR-23 piezoelectricity transducer, with a measurement range of −1 to 1 bar and a total error < 0.25%, located close to the ignition position. A photodiode sensor (type RL-1) is positioned outside the explosion chamber pointing towards the ignition source, which is used to determine the onset of ignition. Signals from the pressure transducer and the photodiode are recorded with 12 bit resolution at a rate of 15 kHz. The process of the flame propagation is visualized by a high-speed digital camera (LaVision Inc.) that is operated at 3000 frames per s with the resolution of 1024 × 1024 pixels, triggered by the photodiode signal. More details of the experimental system can be found in the previous studies.^31,32^ Each experiment was tested at least 3 times, and all the results obtained were highly reproducible. In this study, each curve represents an average based on at least three tests performed for each case.

## 3 Results and discussion

### 3.1 Comparison of explosion characteristics with and without nanoparticles

**Fig. 2:**
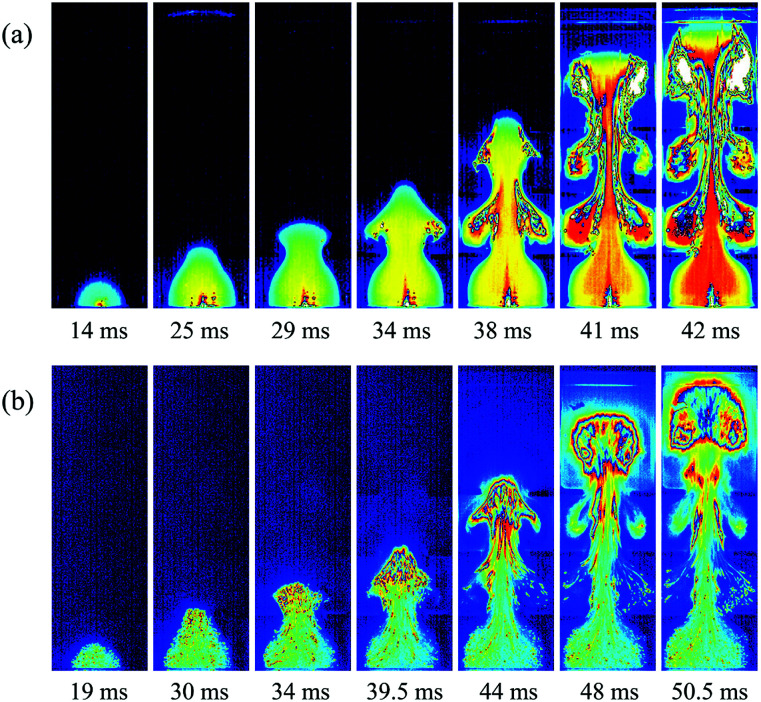
High-speed images of flame propagation: without particle (a) and with particles (b).

To investigate the effects of the nanoparticles on methane–air explosions in the presence of obstacles, the experimental high-speed images of methane–air explosion flame propagation after ignition with and without particles were compared as shown in Fig. 2. Corresponding to similar flame front distances from the bottom end after ignition, typical seven images were selected for each cases. For the case with nanoparticles, Mg(OH)_2_ particles with a averaged 30 nm diameter and 25 g m^−3^ density were considered.

As shown in Fig. 2a, in the case without particles, the flame is found to be spherical from *t* = 0 to 20 ms, propagating radially from the ignition point. At this time, the flame is light blue in color, corresponding to an initial reaction stage. At about 25 ms, the flame is crossing the first pair of obstructions, and the flame front looks like a tongue jetting past the gap between the two parallel obstacles. The flame becomes yellow-colored around the center and light blue near the edge of the flame. Then the flame starts to expand in the transverse direction, and it curls around the vortexes behind the obstacles, which develops into a typical mushroom-like shape. At this stage, flame surface area increases because the flame front consumes the mixture in the vortexes. This same sequence is repeated, with ever-higher velocities, when the flame crosses the second and third pairs of obstacles before it approach the exit end of the chamber. At this stage, the flame is red within the wake region of the three pairs of obstacles, which confirms that the flame structure becomes distorted and turbulent with an increase in flame surface area, causing an amount of unburnt gases to mix with high-temperature burnt gases.

Comparing Fig. 2a with Fig. 2b, it is clear that the addition of particles has a significant impact on the flame propagation of methane–air explosion. At the earlier time (*t* = 19 ms), a similarly spherical flame structure with a light blue color is observed, but it can be seen that some particles already being engulfed within the burned mixture. At 30 ms, the flame front reaches the first pair of obstacles, which is 5 ms later than without particles, illustrating that the nanoparticles can somewhat suppress the flame propagation. When the flame passes sequentially over the three pairs of obstacles, the vortexes behind the obstructions are also generated gradually. However, the flame observed remains generally light blue, indicating that the burning rates in the reaction zone are limited by the suppressing cloud of particles, which are remarkably different for the case without particles. The main reason for this is the fact that the nanoparticle is finer, and the specific particle surface area is larger, so they are easy to contact with and absorb radiant heat near the combustion reaction region.^3^ Especially in the presence of obstacles, the flame–obstacle interaction may cause more particles mix with high-temperature burnt gases at a faster rate, enhancing the suppression effect of the nanoparticles, eventually disrupting or quenching the flame.

**Fig. 3:**
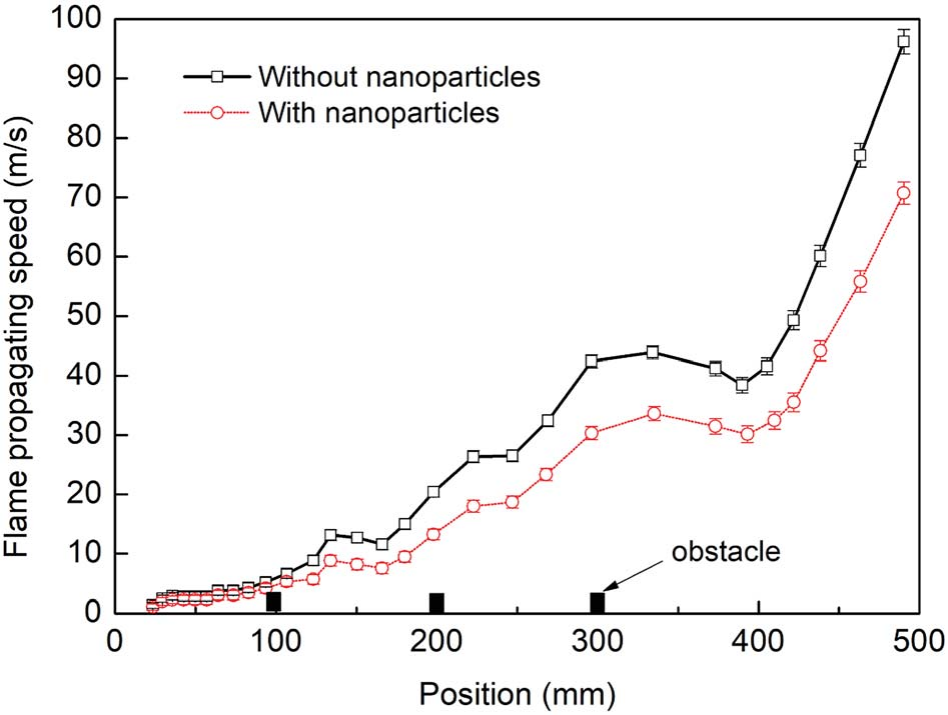
Flame propagating speeds with and without particles.

Fig. 3 presents a comparison of flame speeds plotted against the axial distance from the ignition between the cases with and without particles. The error bars represents the standard deviation in the velocity data measured in the repeated tests. The flame speed is determined at the tip of the flame front far downstream from the ignition end. As shown in the two cases, the similar significant increase in flame speed should be mainly dependent on the interaction of the flame with the three pairs of obstacles. However, for the case where nanoparticles are not applied, the flame speed increases more quickly. The level of flame acceleration as it arrives as the exit of the chamber increases to 96 m s^−1^, while the maximum flame speed is attained about 71 m s^−1^ when sprayed with nanoparticles. The data confirm that when influenced by nanoparticles, the flame acceleration rate decreases significantly.

**Fig. 4:**
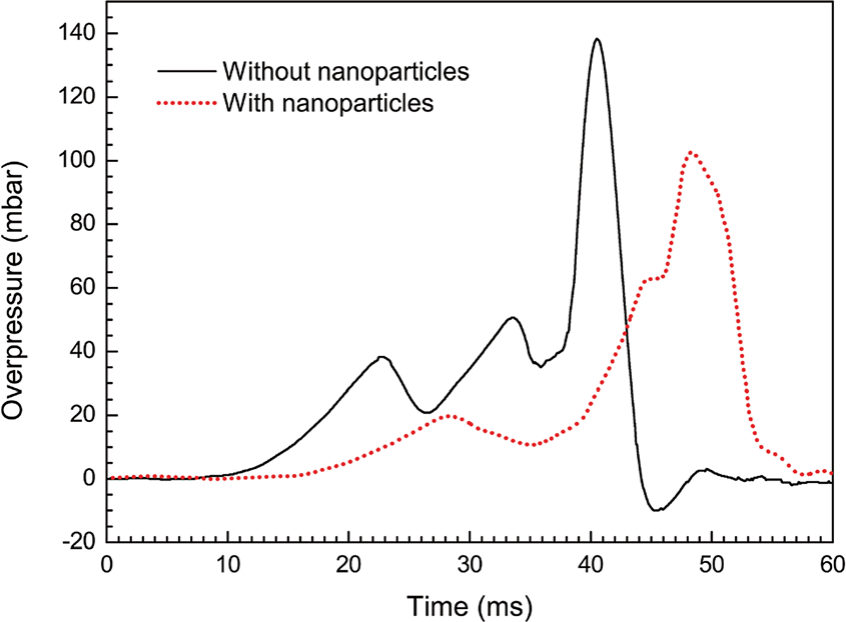
Overpressure histories with and without particles.

Fig. 4 shows overpressure histories from the cases with/without particle additives. In both the curves, three overpressure peaks are found during the explosion processes. From the Fig. 2a and b, it turns out that each time of the pressure peaks corresponds to an acceleration of the flame. For example, the third peak in the case without particles is found at around 40.5 ms after ignition and the flame is passing the third pairs of obstacles at this time. More importantly, we can also see from the Fig. 4 that a higher maximum overpressure of 138 mbar is obtained under the condition of pure methane–air explosion. Comparatively, the magnitude of peak overpressure approximately decreases by 25% in the case with particles, and the time on maximum value occurrence is delayed about 7 ms. It is obvious that just the nanoparticles make explosion overpressure depressed.

### 3.2 Effects of particle density

In this work the effects of particle density, size, and material on flame propagating behavior as well as dynamic overpressure were studied. Note that all cases tested in this section were carried out with three pairs of obstacles. At first, four different particle densities, *i.e.* 25, 50, 100, and 150 g m^−3^, were used respectively, with given averaged particle size (30 nm) and material (Mg(OH)_2_).

**Fig. 5:**
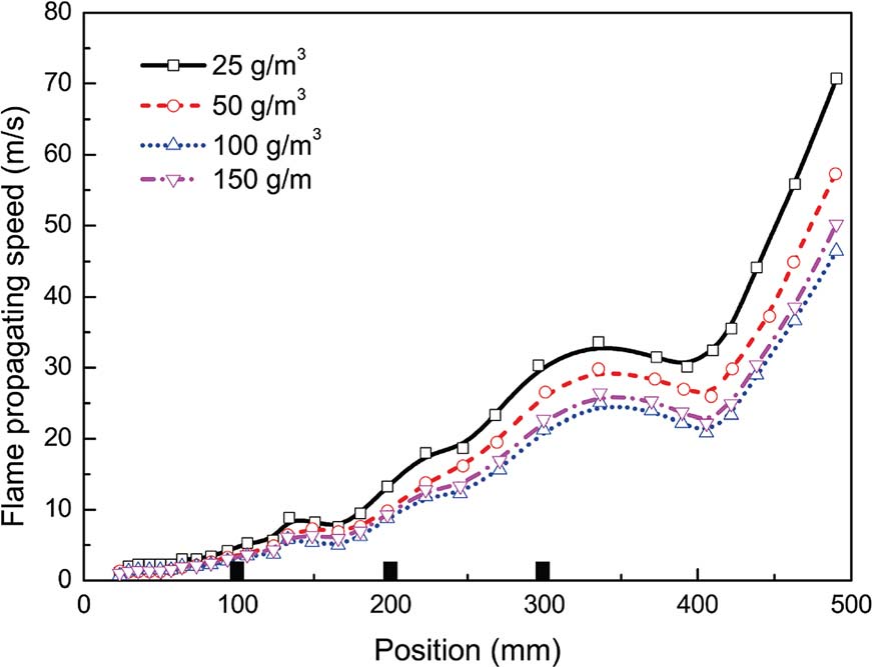
Flame propagating speeds under four particle densities.

**Fig. 6:**
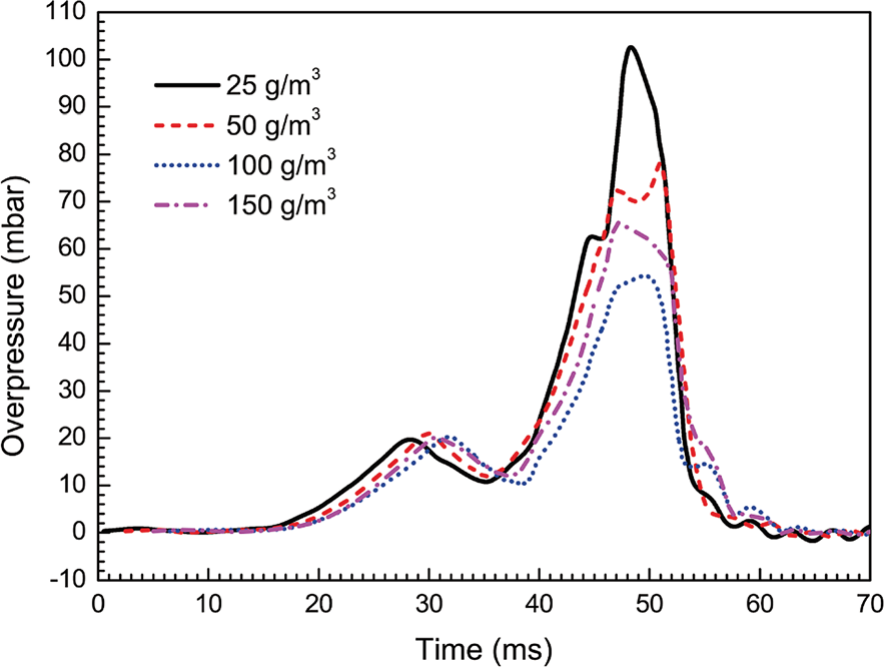
Overpressure transients under four particle densities.

Fig. 5 and 6 present the flame speeds and overpressure transients of methane–air explosion under the four particle densities, respectively. It is can be seen that all the flame propagation speeds in the cases are gradually increased with time induced by the interaction between the flame and the obstacles. In comparison with the reference case (*i.e.* without particles) as shown in Fig. 3, the particle density has an obvious effect on the suppression ability for gas explosion. The data show increasing explosion suppression up to about 100 g m^−3^ particle density. By comparing with the case without particles, the flame propagating speed (47 m s^−1^) and overpressure peak (55 mbar) for the density of 100 g m^−3^ are decreased by about 51% and 60%, respectively. Further increase in particle density up to 150 g m^−3^, however, yields no further increase in the explosion suppression ability. The results are not fully consistent with previous data.^33,34^ It is suggested that nanoparticles are more easily to be aggregated by an attraction force between the particles when the particle density is large enough,^35^ which is obviously different from microparticles.

### 3.3 Effects of particle size

**Fig. 7:**
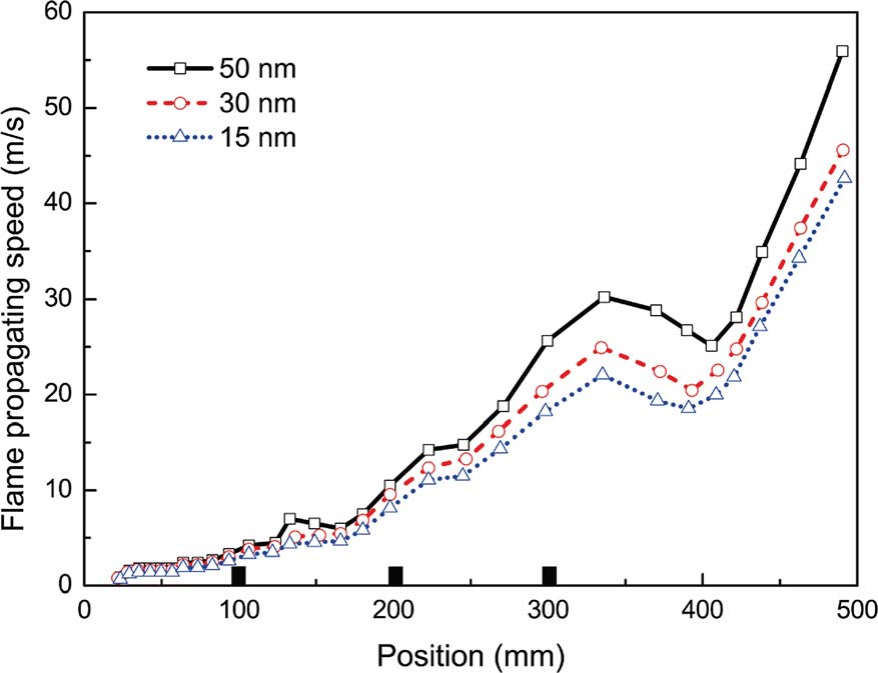
Flame propagating speeds with varying averaged particle sizes.

Then, taking the same density (100 g m^−3^) and the same material (Mg(OH)_2_) of nanoparticles, in the experiments with varying averaged particle sizes, 15, 30 and 50 nm, the observed flame propagating speeds are displayed in Fig. 7. Obviously, no complete suppression of explosion is reached even with the particle diameter at 15 nm, and it also shows that smaller the particles are, the more evident the suppressing effect become.

**Fig. 8:**
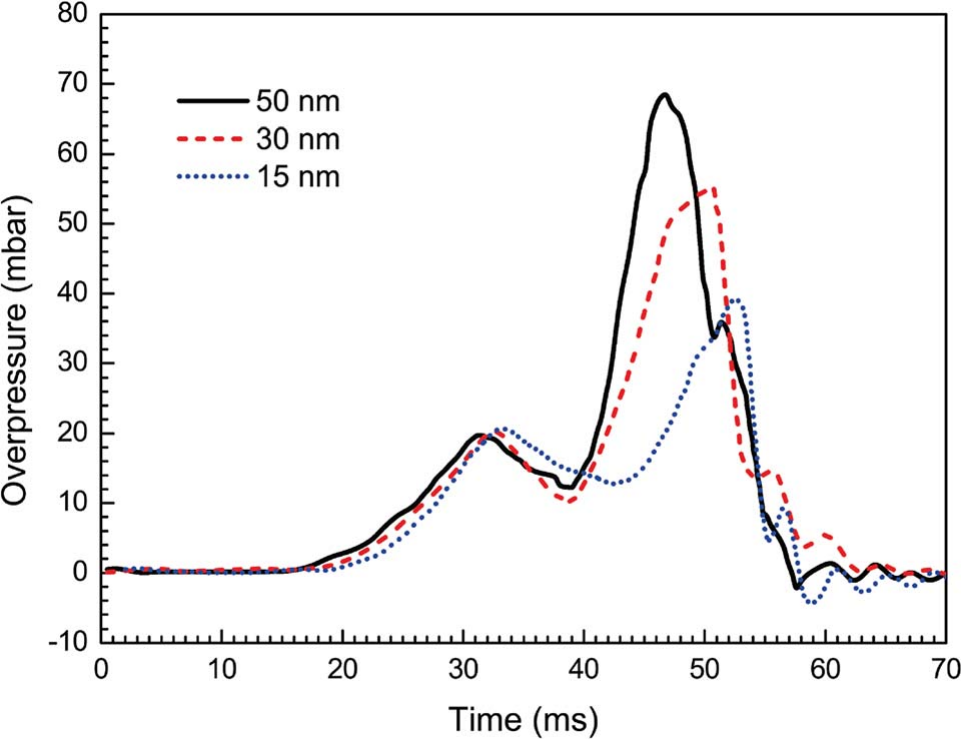
Overpressure histories with varying averaged particle sizes.

Fig. 8 presents the overpressure–time curves for the cases with different particle sizes. It is expected that as the particle size decreases, the peak overpressure decreases, and the time to reach the peak increases. Specifically, the highest overpressure (69 mbar at 47 ms) is obtained for the case with 50 nm size, and the lowest overpressure is obtained for 15 nm. The increase in suppressing effectiveness with a reduction in particle size can be explained by the corresponding increase in particle surface area, which leads to greater radiant heat absorption.^36^ As a result, it is desirable to use as small nanoparticles as possible to improve the efficiency of explosion suppression.

### 3.4 Effects of particle material

Besides Mg(OH)_2_, other particles have been brought into wide use as flame retardants.^21,31,34,37^ In order to further explore the influence of nanoparticle material on suppressing behaviors against methane–air explosion, three different particle materials, *i.e.* Al(OH)_3_, Mg(OH)_2_, and SiO_2_, were used respectively, in which the given particle density (100 g m^−3^) and the averaged size (30 nm) were considered.

**Fig. 9:**
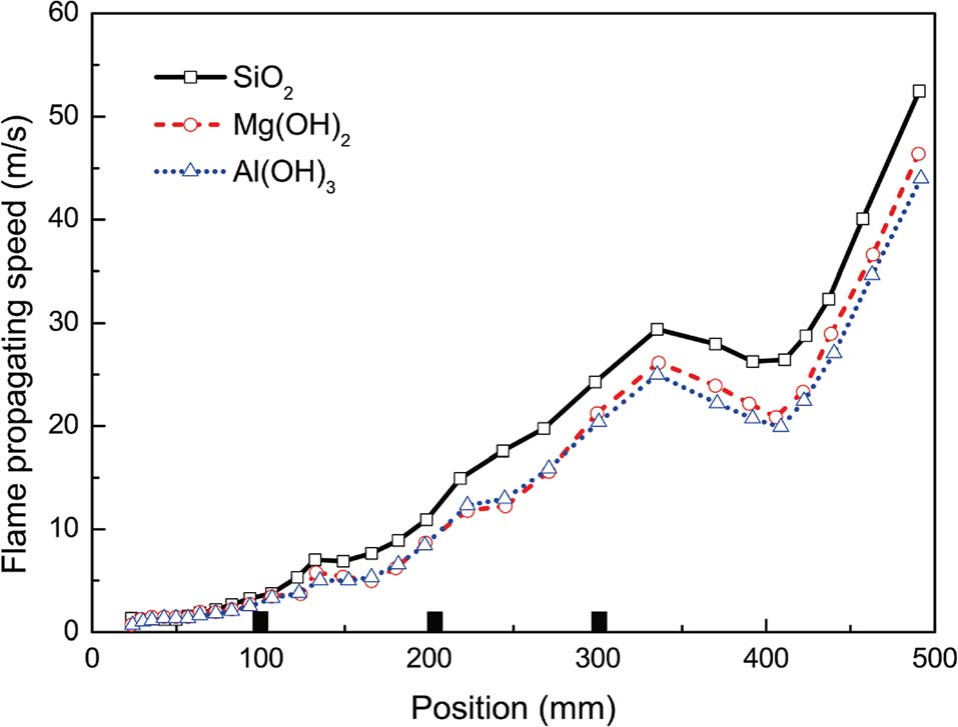
Flame propagating speeds with different particle materials.

**Fig. 10:**
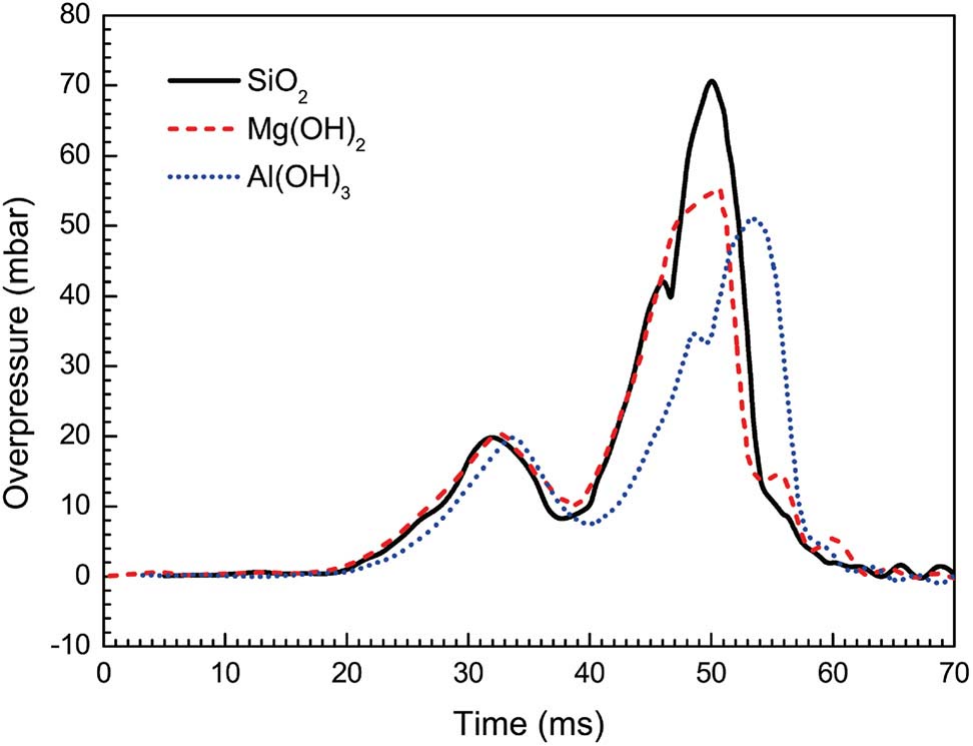
Overpressure histories with different particle materials.

The suppressing effects of these three suppressing agents are compared in Fig. 9 and 10. Totally, the experimental results indicate that all the three kinds of particles can be used to suppress the flame propagation and overpressure. However, Al(OH)_3_ and Mg(OH)_2_ particles suppresses the methane–air explosions even more efficiently than SiO_2_ particles. This phenomenon can be partly but not completely explained by the argument of Wang *et al.*^38^ that the thermal decomposition of the metal hydroxides (Al(OH)_3_ and Mg(OH)_2_) can absorb much of the heat released from the explosion reaction to reduce the flame surface temperature. Meanwhile, water vapor produced by the thermal decomposition results in a decrease of the real methane concentration in the explosion chamber and will decrease the gas burning velocity. In addition, as can be seen from Fig. 9 and 10, Al(OH)_3_ particle has a slightly better inhibiting effect than Mg(OH)_2_. This is believed to be responsible for the fact that Al(OH)_3_ has a lower decomposition temperature range and at the same time can produce more water vapor per unit mass than the latter. At present we cannot determine whether the above results are related to the reduction of free radicals involving in the chemical reaction rate of combustible mixture in the hydroxide clouds. Further studies are needed to resolve this uncertainty, based on this research.

## 4 Conclusions

In this work we experimentally compared in detail flame behaviors as well as overpressure transients with and without nanoparticles, and explored the interactions between flame and inert nanoparticles during gas explosion suppression in an obstructed chamber, which aimed to reveal the effects of inert nanoparticles on gas explosion suppression in the presence of obstacles. Moreover, the effects of density, diameter, and material of nanoparticle on the suppressing behaviors for gas explosion have also been discussed. The practical application of the results is especially the better understanding of the explosion suppression characteristics of nanoparticles. This is useful for both researchers and engineers working in this field. The main results can be summarized as following:

(1) With the nanoparticles addition, the methane–air deflagrating flame remains generally light blue and the burning rates in the reaction zone are limited, although the flame structure is distorted by obstacles. The flame–obstacle interaction makes more particles mix with burnt gases at a faster rate. As a result, the flame acceleration rate and the peak overpressure decrease significantly, while the time of maximum overpressure occurrence is delayed.

(2) Increasing explosion suppression up to about 100 g m^−3^ particle density. However, further increase in particle density up to 150 g m^−3^, yields no further increase in the explosion suppression ability, due to that nanoparticles are more easily to be aggregated if the particle density is large enough. By comparing with the case without particles, the flame propagating speed and overpressure peak for the particle density of 100 g m^−3^ are decreased by about 51% and 60%, respectively.

(3) As the particle size decreases, the suppressing effect is more evident because of the higher specific surface area, which leads to greater radiant heat absorption near the combustion reaction region. Al(OH)_3_, Mg(OH)_2_, and SiO_2_ all can be used to suppress the flame propagation and overpressure. However, the metal hydroxides suppress the methane explosions even more efficiently than SiO_2_ particles, and Al(OH)_3_ particle has a slightly better inhibiting effect than Mg(OH)_2_.

## Conflicts of interest

There are no conflicts to declare.

## Supplementary Material
